# Chemical Magnetoreception: Bird Cryptochrome 1a Is Excited by Blue Light and Forms Long-Lived Radical-Pairs

**DOI:** 10.1371/journal.pone.0001106

**Published:** 2007-10-31

**Authors:** Miriam Liedvogel, Kiminori Maeda, Kevin Henbest, Erik Schleicher, Thomas Simon, Christiane R. Timmel, P. J. Hore, Henrik Mouritsen

**Affiliations:** 1 VW Nachswuchgruppe Animal Navigation, IBU, University of Oldenburg, Oldenburg, Germany; 2 Edward Grey Institute, Department of Zoology, University of Oxford, Oxford, United Kingdom; 3 Inorganic Chemistry Laboratory, University of Oxford, Oxford, United Kingdom; 4 Physical and Theoretical Chemistry Laboratory, University of Oxford, Oxford, United Kingdom; 5 Institut für Experimentalphysik, Freie Universität Berlin, Berlin, Germany; 6 DIARECT AG, Freiburg, Germany; Cairo University, Egypt

## Abstract

Cryptochromes (Cry) have been suggested to form the basis of light-dependent magnetic compass orientation in birds. However, to function as magnetic compass sensors, the cryptochromes of migratory birds must possess a number of key biophysical characteristics. Most importantly, absorption of blue light must produce radical pairs with lifetimes longer than about a microsecond. Cryptochrome 1a (*gw*Cry1a) and the photolyase-homology-region of Cry1 (*gw*Cry1-PHR) from the migratory garden warbler were recombinantly expressed and purified from a baculovirus/*Sf9* cell expression system. Transient absorption measurements show that these flavoproteins are indeed excited by light in the blue spectral range leading to the formation of radicals with millisecond lifetimes. These biophysical characteristics suggest that *gw*Cry1a is ideally suited as a primary light-mediated, radical-pair-based magnetic compass receptor.

## Introduction

Each year millions of migratory birds travel thousands of kilometres between their breeding and wintering grounds. A magnetic compass sense has been shown to play a key role in enabling them to find their way [1]–[3]. But how do migratory birds detect the reference compass direction provided by the Earth's magnetic field? This remains one of the most fascinating unresolved mysteries of sensory biology. From behavioural experiments, we know that the ability of night-migrating birds to perform magnetic compass orientation is affected by the wavelengths of the ambient light [4], [5] and that pinealectomised birds [6] are still able to use their magnetic compass to orient in the laboratory. This led to the suggestion that photochemical reactions in the birds' eyes could produce spin-correlated radical pairs as the primary sensor in a magnetic field-dependent signalling cascade [7], [8], based on the fact that the recombination reactions of spin-correlated radical pairs are known to be magnetic field-sensitive under favourable conditions [9], [10].

Spin-correlated radical pairs are typically the result of a photochemical reaction (e.g. electron transfer) that creates radicals from a molecular precursor that is in either an electronic singlet (S) or triplet (T) state. Under the influence of intramolecular electron-nuclear hyperfine interactions, the radical pair can oscillate between the S and T states, a process known as S↔T interconversion or S↔T mixing. External static magnetic fields can affect the extent and frequency of this process and hence alter the yields of the respective reaction products formed from the S and T radical pairs [10], [11]. In order for a magnetic field to have a substantial effect on the S↔T mixing process, the radical pair needs to be sufficiently long-lived (for an Earth-strength magnetic field, the radical pair lifetime should probably exceed ∼1 µs; [12]). Despite encouraging theoretical considerations [8] and supporting calculations [12], [13], there is no experimental proof as yet that the recombination yields of such radical pair reactions are sensitive to the *orientation* of the pair with respect to the external magnetic field (or indeed that any radical pair reaction responds significantly to a field as weak as the Earth's (∼50 µT)). This directional information is of course of pivotal importance in the discussion of magnetoreception in migratory birds.

Magnetic fields oscillating with frequencies in the MHz range are also known to affect the S↔T interconversion in spin-correlated radical pairs, even in the absence of a static external magnetic field [8], [14]–[16]. The hypothesis that the avian magnetic compass is based on a radical pair mechanism is strongly supported by the observation that magnetic compass orientation of migratory birds can indeed be disturbed by weak radiofrequency magnetic fields with frequencies in the MHz range [17], [18]. However, for this radical-pair hypothesis to be realistic it must be shown that molecules with the required biophysical characteristics actually exist in the eyes of migratory birds. Cryptochromes [19], a class of proteins that exhibit a close homology to photolyases (flavoproteins involved in the repair of UV light-induced DNA damage), were first proposed by Ritz *et al.* [8] as the host molecules for the crucial radical pair cofactors that putatively act as a primary magnetoreceptor. It has recently been demonstrated, using UV/visible and EPR (electron paramagnetic resonance) spectroscopies [20]–[22], that photolyases are able to form long-lived radical-pairs including a flavin radical and a radical derived from a redox-active amino acid. So far it has been shown that cryptochromes exist in the eyes of migratory birds [23], [24], and that at least some cryptochrome-containing cells within the retina are active at night when the birds perform magnetic orientation in the laboratory [23]. Furthermore, a distinct part of the forebrain which primarily processes input from the eye is highly active at night in night-migratory garden warblers (*Sylvia borin*) and European robins (*Erithacus rubecula*) [25], [26]. All these findings are consistent with the hypothesis that magnetic compass detection in migratory birds takes place in the eye and that cryptochromes could be a primary magnetoreceptors. Despite this mounting body of indirect evidence, it has still be to demonstrated convincingly that cryptochrome is directly involved in the avian magnetic compass sense. For instance, several fundamental biophysical characteristics including the absorption spectrum and the formation of long-lived radical pairs have not been experimentally validated for any vertebrate cryptochrome [27]. The aim of the present study is to experimentally explore the biophysical properties of cryptochromes from migratory garden warblers.

## Materials and Methods

### Cloning

For detection and cloning of garden warbler (*gw*) Cry1 transcripts, total RNA was extracted from the retina of a garden warbler performing magnetic orientation behaviour in an orientation cage [28] in the laboratory during the migratory season as described in [23]. The retina was collected immediately after the bird had shown at least 1 h of consistent migratory-restlessness behavior. cDNA synthesis was performed from 450 ng of RNA with Revert AID H^−^First-Strand cDNA synthesis kit (MBI Fermentas) according to the manufacturer's protocol.

Hot start PCR amplification reactions were performed in 25 µl total reaction volume containing 1 µl of the cDNA synthesis reaction as template, 1× reaction buffer (SAWADY/Amersham-Pharmacia, depending on the DNA polymerase used), 0.25 mM dNTPs (Pharmacia Biotech), of each primer 1.25 pmol/µl PCR volume, 1 unit of *Pwo* polymerase (SAWADY) or 2 units of *Taq* polymerase (Amersham-Pharmacia). The PCR reaction was carried out in a MWG Primus25 thermocycler. The temperature profile for all hot-start PCR reactions was as follows: first denaturation 3 min 94°C; 45 cycles of 45 s at 94°C, 45 s annealing at 54°C and 40 s extension at 72°C (the duration of extension phase depended on the expected size of the amplified fragment and the enzyme used; the extension step comprised 1 min/kb DNA for amplification with *Taq* polymerase and 2 min/kb DNA for *Pwo* amplification); the last cycle was extended to 5 min at 72°C to ensure completion of the final extension. The full length sequence of *gw*Cry1a (GenBank accession no. AJ632120) was reconstructed as described in Mouritsen *et al.* [23].

To probe for potential influences of the C-terminal extension of *gw*Cry1a in the reaction process, we additionally expressed the photolyase homology region (510 aa) of *gw*Cry1a (Cry1-PHR). The PHR lies within the N-terminal domain, which is identical for both *gw*Cry1a and *gw*Cry1b (GenBank accession no. DQ838738), is similar to photolyases and highly conserved in all members of the photolyase/cryptochrome superfamily [29], [30]. For expression of *gw*Cry1-PHR the N-terminal 510 amino acids were chosen based on the structural analysis of *A. thaliana* Cry1-PHR [29] and the lack of homology between garden warbler and *A. thaliana* Cry1 beyond amino acid residue 510. A C-terminal fragment for *gw*Cry1-PHR was PCR-amplified from a cloned *gw*Cry1a template with the primer pair: DSP^1PHR^ (*p741*), 5′-TAGTTCCTTCTTTCAGCAGTTT-3′; USP^1PHR^ (*p742*), 5′-AGCAAGCGGCCGCTTATCAA GTTGCAAGAAGACCCAGTCCT-3′. The amplified fragment (320 bp) was digested with *BstXI/NotI* (resulting in a 107 bp fragment) and cloned with a 1408 bp N-terminal fragment isolated from a full-length *gw*Cry1a plasmid by *NcoI/partial BstXI* digest of the full length *gw*Cry1a plasmid.

Coding regions for *gw*Cry1a and *gw*Cry1-PHR proteins were subcloned into a pVL1392-derived baculovirus transfervector to allow for subsequent recombinant expression of the target proteins in a *Sf9*/baculovirus expression system. For selective purification by means of immobilised metal affinity chromatography (IMAC) matrices, we expressed the genes as fusions with a vector-encoded N-terminal (His)_6_-tag. The full length sequence of *gw*Cry1a (1866 bp) was subcloned into the polylinker region of the pDIA92B-His transfervector by *SmaI/NotI* digest. The PHR region of *gw*Cry1 (1536 bp) was subcloned into the polylinker region via *NcoI/NotI* digest. Recombinant baculoviruses were generated by cotransfection of transfer vectors and baculoviral genomic DNA and clonal isolation of *gw*Cry1 protein-expressing baculoviruses according to standard methods [31], [32].

### Recombinant expression

To characterise the molecular properties of *gw*Cry1a and *gw*Cry1-PHR proteins, we expressed the proteins in *Sf9* cells transfected with the corresponding recombinant baculovirus. Infected cells were incubated in ExCell420 culture medium (JRH) supplemented with 25 µM each of FAD and FMN (Sigma) in spinner flasks (0.6 l culture volume) for 66 h (±4 h) at 27°C. Cell pellets were harvested, washed in PBS and quick-frozen in liquid nitrogen.

### Purification

*gw*Cry1a (628 aa; ∼62 kDa) and *gw*Cry1-PHR (510 aa; ∼56 kDa) were expressed as soluble fusion proteins and purified to near homogeneity by immobilized metal affinity chromatography IMAC) in 20 mM HEPES buffer (pH 7.4) containing 500 mM NaCl and 50 µM FAD. Recombinant *gw*Cry1a was bound to the Chelating Sepharose matrix and unspecifically bound contaminants were washed away with 20 mM HEPES buffer containing 500 mM NaCl and 50 µM FAD (pH 7.4). The column-bound protein was denatured by a linear gradient (10 column volumes) of 0 M to 6 M urea in 20 mM HEPES buffer containing 50 µM FAD, renatured by a “reversed” linear gradient from 6 M to 0 M urea in 20 mM HEPES 50 µM FAD buffer, and finally eluted by increasing imidazole concentration. Samples were concentrated in Vivaspin contractor columns (membrane: 30,000 MWCO) (Sartorius). Excess unbound (free) flavin was eliminated by a final desalting step using HiTrap 5 ml columns (Amersham Bioscience). Samples were stored in 20% glycerol at a final protein concentration of 15–25 µM, shock frozen in liquid nitrogen and stored at −80°C.

The quality of the purified proteins was analysed by SDS-PAGE and Western blot analyses using standard procedures. On PVDF membrane (Pall) Western blots, *gw*Cry1a protein was probed by goat polyclonal antibody α(anti)-Cry1 (A-20) *sc-5953* (Santa Cruz Biotechnology), and *gw*Cry1-PHR was detected by a commercially available mouse monoclonal α-His tag antibody (PentaHis antibody, Qiagen); the primary antibodies were detected by appropriate alkaline phosphatase-conjugated secondary antibodies and nitroblue tetrazolium/bromo-chloro-indoxyl phosphate staining on the membrane.

Samples were concentrated by ultrafiltration through 30 kDa microconcentrators (Millipore, Billerica, MA, USA) at 4°C. The flowthrough was periodically monitored spectroscopically: concentrations of free flavin were below the detection limit of 1 µM.

### UV/visible spectral analysis

Purified *gw*Cry1a and *gw*Cry1-PHR, both containing FAD in its fully oxidised redox state, were supplemented with 5 mM DTT, deoxygenated and illuminated at 290 K with blue light (Halolux 30HL, Streppel, Wermelskirchen-Tente, Germany) selected with a 420–470 nm band filter (Schott, Mainz). Reoxidation was performed under aerobic conditions in the dark. The concentration of protein-bound flavin was estimated based on its absorbance at 450 nm (ε_450_ = 11.3×10^4^ M^−1^ cm^−1^). The concentrations of *gw*Cry1-PHR and *gw*Cry1a holoproteins, with FAD bound, were 7 µM and 4 µM respectively.

### Transient absorption experiments

**Figure 1:**
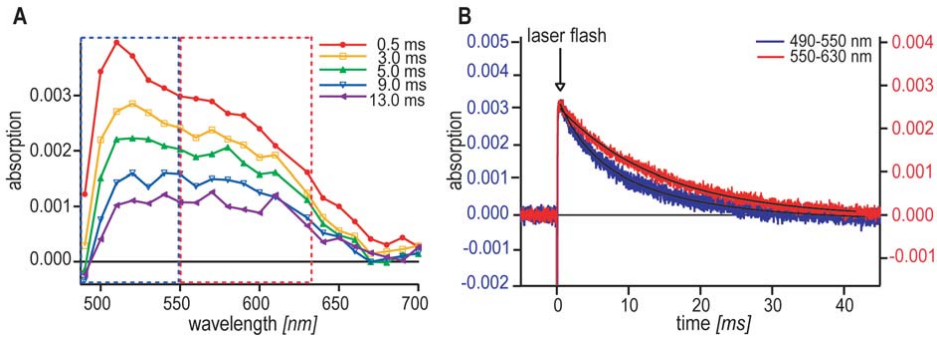
Transient absorption spectra for *gw*Cry1a. A Transient absorption spectra for *gw*Cry1a as a function of wavelength (monitoring beam) proves that radical pairs are produced in cryptochromes of a migratory bird. The five graphs reflect the optical properties of the sample at different times, *t*_delay_, after the laser flash. The amplitude of the signals depends on the protein concentration. The spectra were obtained by averaging two time profiles (cf. Fig. 1B) centred at *t*_delay_ using a time window of 500 µs. The variation of the absorption signal is about 5%, which is estimated from the data at 0.5 ms from 500 to 600 nm. For a detailed analysis of the spectra, see main text. B Transient absorption time profiles for *gw*Cry1a. A laser flash (indicated by the arrow) is given at time = 0. The transient signal is obtained by averaging 7 time-profiles for the 490–550 nm wavelength region (corresponding to the optical features framed in blue in Fig. 1A) in 10 nm steps (blue curve, left scale) and 9 time-profiles for the 550–630 nm region (corresponding to the optical features framed in red in Fig. 1A) in 10 nm steps (red, graph, right scale), respectively. Solid lines show the fitted single or double exponential decays. The laser flash leads to radical formation as reflected by positive changes in the absorption signal. Significant radical concentrations are detectable up to ∼25 ms. For further discussion, see main text.

Solutions of proteins (concentrations in the range 15–25 µM) in 20 mM HEPES buffer and 20% glycerol at 270 K were photoexcited at 355 nm using a Nd-YAG laser (5 ns, 5 mJ/pulse). The monitoring beam was provided by a tungsten lamp (150 W). Light from the sample was fed to a monochromator (Newport model 77250) before entering a photomultiplier tube (Hamamatsu R-928). The time-resolution of the measurement was 20 µs. The signals presented in Figure 1 are averages of 2 measurements.

### NMR spectroscopy

600 MHz ^1^H NMR spectra of the ∼20 µM *gw*Cry1a samples used for the transient absorption experiments were recorded on a Varian Inova 600 (14.1 T) NMR spectrometer to confirm the absence of significant amounts of free flavin (see Supporting Information (Text S1, Fig. S1)).

Animal husbandry as well as all experiments and animal procedures were approved by the Animal Care and Use Committees of the Bezirksregierung Weser-Ems (Oldenburg, Germany).

## Results

### Garden warbler cryptochrome (gwCry1a) absorbs blue light

**Figure 2:**
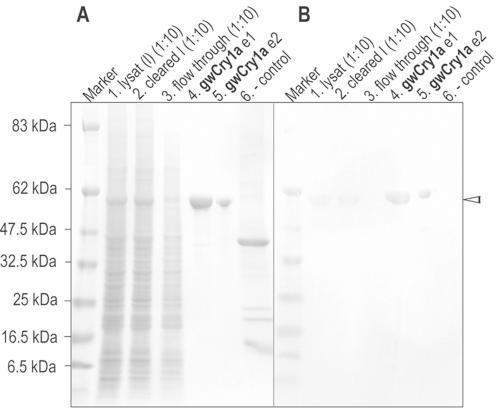
SDS-PAGE of recombinantly expressed garden warbler cryptochrome (shown for *gw*Cry1a protein). A Coomassie stained SDS gel, and B Western-blot (with α-Cry1 antiserum from immunised goat), both showing identical fractions of the IMAC purification process. *gw*Cry1a (628 aa) is recognisable as a distinct band at ∼62 kDa in both the coomassie stained SDS gel and on the Western-blot (indicated by the arrowhead to the right). *1: gw*Cry1a lysate (protein extract from insect cell pellet, 1∶10 dilution); *2: gw*Cry1a cleared lysate (centrifuged and filtered lysate, 1∶10 dilution); *3: gw*Cry1a flow through (after sample loading on column, 1∶10 dilution); *4: gw*Cry1a elution step1 (e1) [75% imidazole]; *5: gw*Cry1a elution step2 (e2) [100% imidazole] protein quantity for e2 is 5 µg; *6:* negative control (recombinantly expressed human La/SSB autoantigen; ∼48 kDa) confirms the specificity of the antibody.

**Figure 3:**
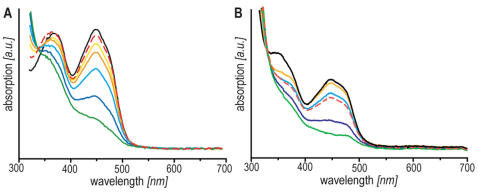
Optical spectroscopy of cryptochrome holoprotein from migratory garden warblers. A *gw*Cry1-PHR, illumination time: 0 s (black), 40 s (yellow), 80 s (orange), 130 s (light blue), 190 s (blue) and 340 s (green), respectively. B *gw*Cry1a, illumination time: 0 s (black), 90 s (yellow), 150 s (orange), 240 s (light blue), 360 s (blue) and 540 s (green), respectively. Both samples were illuminated under anaerobic conditions as indicated in the Methods section. Reoxidation was achieved by bubbling air into the optical cuvette (red dashed lines).

To determine the visible absorption spectra of garden warbler cryptochromes, we recombinantly expressed and purified Cry1a and the photolyase homology region of the migratory warbler (shown for *gw*Cry1a in Fig. 2). The absorption spectra of purified *gw*Cry1a and *gw*Cry1-PHR proteins are shown in Fig. 3. FAD (flavin adenine dinucleotide) cofactors show characteristic optical absorption properties in all three of their biologically relevant redox states: fully reduced (FADH^−^ or FADH_2_), semiquinone radical (FAD^•−^ or FADH^•^), and fully oxidised (FAD_ox_). Both cryptochrome constructs were isolated in a yellow form characteristic of a flavoprotein with a fully oxidised cofactor, FAD_ox_. *gw*Cry1a and *gw*Cry1-PHR with bound FAD_ox_ both have absorption maxima at 450 and 366 nm representing respectively the S_0_→S_1_ and S_0_→S_2_ transitions of the flavin cofactor. Members of the photolyase family often bind a pterin or deazaflavin in addition to the FAD [for reviews see 30], [33], [34]: there was no evidence of a second chromophore in either of the samples. The spectra resemble those of other flavoproteins [35] except that the vibrational structure of the 450 nm band is not pronounced; we return to this point below.

Illumination of *gw*Cry1a and *gw*Cry1-PHR with blue light (420<λ<470 nm) attenuates both bands (Fig. 3). This behaviour is characteristic of the photolyase/cryptochrome superfamily [e.g. 36], [37], [38]. After illumination in the presence of dithiothreitol (DTT; as an exogenous electron donor), the fully (two-electron) reduced FADH^−^ form of the flavin was formed (Fig. 3), exhibiting a broad absorption band around 360 nm. In contrast to other members of the photolyase/cryptochrome family, however, flavin radicals (exhibiting absorption maxima at around 580 and 620 nm) do not accumulate to a significant extent in *gw*Cry1a and *gw*Cry-PHR. Moreover, no significant radical formation is observed while reoxidising the proteins under aerobic conditions. The reason could be either a small energy difference between the radical and the fully reduced form of the FAD, or, more likely, that radical disproportionation is occurring on a fast timescale [20].

### Garden warbler cryptochrome forms long-lived radical-pairs

To test whether *gw*Cry1a forms radical-pairs upon photoexcitation and to investigate whether they have sufficient lifetimes for the putative geomagnetic field effects to take place, we recorded transient absorption spectra for *gw*Cry1a protein using laser flash photolysis at 355 nm. The identity of the transient photo-intermediates formed from *gw*Cry1a was investigated *via* their time-resolved absorption characteristics (Fig. 1). Figure 1A shows the transient absorption spectra obtained for *gw*Cry1a as a function of both the time delay between the laser flash and the 500 µs monitoring window (*t*_delay_ = 0.5, 3, 5, 9, 13 ms) and the wavelength of the monitoring light (horizontal axis). The spectra show that garden warbler cryptochrome forms long-lived radical pairs and that this radical-pair formation is strongly dependent on both wavelength and time, with a pronounced absorption peak around 510 nm at early times (*t*_delay_ = 0.5 ms). It is clear from a comparison of the five spectra, that this peak (blue dotted frame) has a shorter lifetime than the features at longer wavelengths (red dotted frame). Figure 1B shows this behaviour in a more quantitative fashion. Here, the absorption of the sample is detected as a function of time after the laser flash for the two wavelength regions (blue curve: 490–550 nm; red curve: 550–630 nm). The shorter wavelength decay can only be modelled satisfactorily as a double exponential decay with lifetimes of 4 ms and 14 ms, while the longer wavelength decay can be accurately modelled as a single exponential with a lifetime of 14 ms.

The absence of pronounced vibrational structure around 450 nm in Fig. 3 may, at first glance, suggest a free flavin molecule rather than a flavin cofactor bound to a photolyase or a cryptochrome. The protein purification protocol used (see Material and Methods and Supporting Information (Text S1, Fig. S1, Fig. S2)) makes it is very unlikely that there were any significant amounts of free flavin left in our samples. Nevertheless, considering the form of the recorded spectra, it is important to demonstrate unequivocally that the transient absorption data (Fig. 1) cannot originate from radicals formed from free flavin molecules. We therefore performed a number of control experiments including NMR spectroscopy and transient absorption measurements on solutions of FAD and FMN (flavin mononucleotide) with either tryptophan or hen lysozyme, a ∼14 kDa protein containing two solvent-accessible tryptophan residues that are known to be reactive with photoexcited flavins [39], [40]. As described in detail in the Supporting Information (Text S1, Fig. S1, Fig. S2), these control experiments include NMR spectra that show no detectable amount of free flavin in the samples; additional ultra-filtration steps after protein purification that yield no detectable amount of free flavin; transient absorption measurements showing that free flavin results in signals that are one to two orders of magnitude weaker than the cryptochrome signals presented here and that free flavin radicals are about two orders of magnitude shorter lived than those observed for garden warbler cryptochrome under the same conditions. These control experiments unequivocally establish that the data presented here do indeed result from photo-induced intra-protein electron transfer between garden warbler cryptochrome and its bound flavin cofactor.

## Discussion

We have experimentally verified several key assumptions crucial for the theoretical concept of light-mediated magnetoreception in birds [7], [8]. We succeeded in expressing *gw*Cry1a, which occurs in the retinae of magnetically orienting night-migratory garden warblers [23], and its highly conserved N-terminal PHR domain as holoproteins (with their flavin cofactor bound). The absorption spectra of flavoproteins are dependent on the redox status of the bound flavin cofactor, which in turn is influenced both by the apoprotein and by the redox environment [41]. *gwCry1a* and *gw*Cry1-PHR purified from insect cells both contained the flavin cofactor in the fully oxidised state (Fig. 3), as was also found for isolated *At*Cry1 from the plant *Arabidopsis thaliana* [36]. In isolated photolyases, however, the flavin cofactor is usually bound in a mixture of reduced and semiquinone states [e.g. 42], [43]. The absorption spectrum of *gw*Cry1a fits with the observations that songbirds, including garden warblers, are able to orient well using only their magnetic compass under blue and turquoise light, whereas they seem challenged doing this under yellow and red light [2], [4], [5]. The fact that songbirds are well-oriented under green light (565 nm) is not consistent with the absorption spectra of the fully oxidised form of garden warbler cryptochrome. However, a second longer-wavelength receptor could well be involved [e.g. 5], [44,45].

Using a laser flash photolysis experiment, we were able to prove that illumination of *gw*Cry1a (at 355 nm) produces radical pair species with lifetimes of the order of milliseconds. This is a crucial finding as the evolution of a magnetic field effect relies on the existence of radical pairs that live long enough for the field to affect the S↔T mixing processes. For a magnetic field as weak as that of the Earth, the lifetime should exceed at least a microsecond; a condition clearly fulfilled by the radical species produced in the cryptochromes investigated here which live about five orders of magnitude longer than the minimum required lifetime. Although radical pairs with appropriate lifetimes have been detected in *At*Cry1 [37], and now in the migratory garden warbler, it has still to be demonstrated directly that magnetic fields as weak as the Earth's (∼50 µT) can affect cryptochrome signalling in any species.

**Figure 4:**
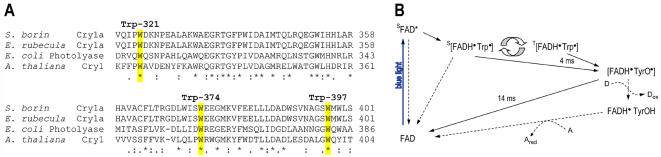
Sequence alignment between different members and species within the cryptochrome/photolyase protein family. A Sequence alignment of Cry1a of two migratory songbird species *Sylvia borin* (Garden warbler; GenBank accession no. AJ632120), *Erithacus rubecula* (European robin; AY585716), Cry1 of *Arabidopsis thaliana* (mouse-ear cress; Q43125) and DNA photolyase of *Escherichia coli* (P00914). The alignment shows that all three members of the Trp-triad electron transfer chain known to be involved in intraprotein electron transfer during signalling through photoreduction in both *E. coli* photolyse and *At*Cry1 are clearly present in the Cry1a sequences of both migratory bird species (Trp-397, Trp-374, Trp-321). B Reaction scheme for *gw*Cry1a (adapted from a very similar scheme for *At*Cry1 proposed by Giovani *et al.* [36]). Processes directly observed here are represented by solid arrows. Other steps are indicated by dashed arrows [36]. The excited state of the FAD is assumed here to be a *singlet*; an almost identical scheme would result if, instead, *triplet* FAD were assumed to be the photoactive excited state. Singlet-triplet interconversion in the [FADH^•^ Trp^•^] radical pair is indicated by the curved block arrows. D and A are respectively an external electron donor and acceptor.

The three members of the Trp-triad electron transfer chain in *At*Cry1 (Trp-400, Trp-377, Trp-324) that are involved in signalling through photoreduction [37], [46] are highly conserved amongst members of the photolyase/cryptochrome family and clearly present (Trp-397, Trp-374, Trp-321) in the Cry1a sequences of both migratory garden warbler and European robin (*Erithacus rubecula*) cryptochromes, (Fig. 4A). On this basis, and by analogy with the recently proposed reaction scheme for *At*Cry1 [37] (Fig. 4B) we interpret the results as follows. Illumination of the sample by the laser pulse is followed by rapid formation of a primary radical pair consisting of a flavin radical, FADH^•^ (broad absorption from 500 to 650 nm, 5 ms half-life in *At*Cry1, 14 ms lifetime in *gw*Cry1a) and a tryptophanyl radical, Trp^•^ (narrow component from 500 to 550 nm, 1 ms half-life in *At*Cry1, 4 ms lifetime in *gw*Cry1a). This process may be followed by an electron transfer from a tyrosine residue to the Trp^•^ to produce a tyrosyl radical, TyrO^•^, accompanied by a pronounced positive contribution to the transient absorption spectrum in the 460–560 nm range. Giovani *et al.* [37] also observe two slow components (half-lives of 5 ms and > 100 ms, respectively) corresponding to the FADH^•^→TyrO^•^ back electron transfer. The similarities in the two time constants (for the TyrOH→Trp^•^ and FADH^•^→TyrO^•^ electron transfers) strongly indicates that *gw*Cry1a and *At*Cry1 have very similar responses to illumination. The differences in the time constants are probably due to the different nature of the two cryptochromes, and hence the environments of the cofactors, and to the different temperatures of the two measurements (270 K here, 285 K in [37]). The pronounced feature at 510 nm in the transient absorption experiments at *t*_delay_ = 0.5 ms (red trace in Fig. 1A) is most likely attributable to the TyrOH→Trp^•^ electron transfer, again in accordance with the findings of Giovani *et al.* [37].

The methods used to purify cryptochrome of the migratory garden warbler together with the NMR measurements and transient absorption control experiments (see Supporting Information, (Text S1, Fig. S1, Fig. S2)) all indicate strongly that the spectroscopic data presented here are due to photo-induced electron transfer in garden warbler cryptochrome. Nevertheless, the flavin absorption band of our sample does bear a closer resemblance to that of free flavin than that of previously published members of the photolyase/cryptochrome superfamily. Given that there is no significant amount of free flavin present, this suggests that either the flavin is incorrectly bound to the apo-protein, e.g. in a non-native binding site, or that it is in the native binding pocket but, perhaps, rather loosely bound. If the former were true, then it is highly unlikely that there would be aromatic amino acid residues close enough to the flavin to undergo photoinduced electron transfer to give long-lived radicals in high yield, as observed. There could be a nearby tryptophan or tyrosine residue that might donate an electron to the flavin excited state, but such a radical pair would almost certainly be as short-lived with respect to back electron transfer as to be undetectable in our experiments. To get long-lived radicals by intramolecular electron transfer, there must be the possibility of sequential electron transfers as occurs with the Trp-triad in photolyase [47], [48]. It seems highly unlikely that a non-natively bound flavin would, by chance, be close to a series of electron donors at just the right separations and orientations to allow efficient charge separation and energy stabilization and to result in lifetimes so similar to those measured by Giovani *et al*. [37]. By elimination, therefore, we are left with the conclusion that the flavin is bound in the correct site and with the correct orientation to undergo sequential electron transfer reactions with aromatic amino acid sidechains in the protein. The absence of more pronounced vibrational structure in the absorption spectrum seems to be a characteristic of the holo-form of *gw*Cry1. Why it has this unusual property will be a subject of future work.

Crucially, the work presented here proves that cryptochromes of a migratory bird absorb light leading, in *gw*Cry1a, to formation of radical species that live about five orders of magnitude longer than the ∼1 µs theoretically required to elicit significant Earth-strength magnetic field effects. It is important to realise, however, that the radical pairs do not only have to be long lived, but their spin-correlation has also to persist for at least ∼1 µs. This issue can be addressed by time-resolved EPR and by measurements of the magnetic field-sensitivity of the radical pair lifetime; both experiments are challenging because of the low quantum yield of radical pair formation [37]. So far, no animal cryptochrome has been expressed and isolated as a holoprotein in sufficiently high concentration to perform these additional analyses. Furthermore, the cryptochromes have to be restricted in their motion in order for any potential magnetic field effect to encode directional information. In any case, our observation of very long-lived photoinduced radical pairs in a migratory bird cryprochrome considerably strengthens the hypothesis of cryptochromes as the primary magnetoreceptor molecules in the avian magnetic compass [8], [49].

## Supporting Information

Text S1 NMR measurements and transient absorption control experiments.
(0.04 MB DOC)

Figure S1 Aromatic region of the 600 MHz 1H NMR spectrum of *gw*CRY1a protein. Aromatic region of the 600 MHz 1H NMR spectrum of the CRY1a sample used for the transient absorption experiments before (blue) and after (red) addition of 20 µM FAD. Note the additional signals from FAD in the latter spectrum and their absence in the former.
(3.63 MB TIF)

Figure S2 Transient absorption signals at 550 nm for garden warbler cryptochrome and equimolar mixtures of FAD with hen lysozyme. Transient absorption signals at 550 nm recorded for aqueous solutions of 20 µM cryptochrome (purple) and for equimolar mixtures (20, 40 and 80 µM) of FAD with hen lysozyme (blue, orange and red, respectively). The abscissa is time (µs); the ordinate is absorbance. Note the much stronger and much longer lived signal from flavin radicals in the case of the cryptochrome compared to that observed for reactions of free flavin with lysozyme.
(4.19 MB TIF)
