# Supplementary material for: Chemical Magnetoreception: Bird Cryptochrome 1a Is Excited by Blue Light and Forms Long-Lived Radical-Pairs
Source: PLoS One. 2007 Oct 31;2(10):e1106. doi: 10.1371/journal.pone.0001106 (PMC2040520; doi:10.1371/journal.pone.0001106)
Supplement: Text S1 — NMR measurements and transient absorption control experiments. (0.04 MB DOC) [file pone.0001106.s001.doc]

**Supporting Information**

**Chemical Magnetoreception: Bird Cryptochrome 1a Is Excited by Blue Light and Forms Long-Lived Radical-Pairs**

Miriam Liedvogel, Kiminori Maeda, Kevin Henbest, Erik Schleicher, Thomas Simon, Christiane R. Timmel, P. J. Hore & Henrik Mouritsen

As there is no published assay for cryptochromes, the following procedures were used to ensure correct incorporation of the flavin cofactor and to remove all traces of free flavin. Excess unbound flavin was eliminated by a final desalting step using HiTrap 5 ml columns (Amersham Bioscience), followed by an additional concentration step by ultrafiltration through 30 kDa microconcentrators (Millipore, Billerica, MA, USA), during which the flowthrough was periodically monitored spectroscopically: concentrations of free flavin were below the detection limit of 1 μM.

***1. Determination of an upper limit on the free FAD concentration in gwCry1a samples by NMR spectroscopy.***

Figure S1 shows the aromatic region of the 600 MHz ^1^H NMR spectrum of the sample of recombinantly expressed Cry1a of migratory garden warbler used for the transient absorption experiments before (blue) and after (red) addition of 20 μM FAD. Any significant amount of free flavin would have been clearly detectable in the form of narrow resonances at characteristic chemical shift positions superimposed on the broad envelope of signals from the protein. Resolved resonances from protein-bound flavin are not expected to be observable because of the line broadening that would result from the slow molecular tumbling of the protein. No narrow resonances at the chemical shift positions of FAD protons were detected, allowing us to put an upper limit on the concentration of free flavin of ~2μM. Given the measures taken to exclude free flavin from the protein samples (see Experimental Procedures section of the paper) we believe the concentration of free flavin is considerably less than 2 μM. See **Figure S1**.

[**Figure Legend Fig. S1**] Aromatic region of the 600 MHz 1H NMR spectrum of the CRY1a sample used for the transient absorption experiments before (blue) and after (red) addition of 20 µM FAD. Note the additional signals from FAD in the latter spectrum and their absence in the former.

***2. Control experiments to demonstrate that the reported transient absorption data cannot be due to free flavin****.*

***(a) Intensity of transient absorption signals.***

Even though a sub-micromolar concentration of free flavin is guaranteed by the methods used to prepare the Cry1 samples for the optical spectroscopy experiments, and to verify their composition, it is important to demonstrate convincingly that the transient absorption data reported here (Fig. 1) cannot arise from photochemical reactions of free flavin molecules with surface aromatic amino acid residues in the protein. Accordingly, we have recorded transient absorption signals for reactions of FAD and FMN with tryptophan and with surface tryptophan residues in hen lysozyme.

Free flavins undergo photoinduced electron transfer reactions with three of the four aromatic amino acids with rate constants in the order *k*(Trp) > *k(*Tyr) >> *k*(His) [S1-S3]. There is no reaction with phenylalanine. We have therefore performed flash photolysis experiments on flavins in the presence of tryptophan, the most reactive of the three.

Using ~20 μM equimolar solutions of FAD or FMN and tryptophan, the initial absorbance at 550 nm, which is the maximum of the flavin radical absorption band, is ~2 × 10^−4^. The extinction coefficient of flavin radicals at this wavelength is ~2000 M^−1^ cm^−1^ and the path length in the transient absorption experiment was 0.3 cm. The initial radical concentration in the FAD/Trp and FMN/Trp experiments is therefore ~2× 10^−4^/(0.3 × 2000) ≈ 0.3 μM. Similar absorbances, and therefore similar initial concentrations, were found (Fig. S2) in experiments on equimolar mixtures of FAD and lysozyme (a ~14 kDa protein containing two solvent accessible tryptophan residues, W62 and W123, that are known to be reactive with photoexcited flavins [S4, S5]).

The 550 nm absorbance measured for the 20 μM Cry sample was 3 × 10^−3^, corresponding to an initial radical concentration of 5 μM. This is an *order of magnitude larger* than found in any of the three control experiments (Fig. S2). Had this signal been due to 1 μM free flavin contaminating a 20 μM apo-Cry sample, we would have seen an initial absorbance of at most ~2 × 10^−4^/20 = 10^−5^, i.e. two orders of magnitude smaller than actually observed.

The transient absorption signals are thus much stronger than expected for the reaction of free flavin with surface aromatic amino acid residues. The signals reported in Fig. 1 are only weak in absolute terms because the protein concentration is low.

***(b) Kinetics of transient absorption signals.***

Further evidence that free flavin cannot be responsible for the observed transient absorption signals (Fig. 1) comes from the lifetimes of the absorption signals measured for the photoreaction of FAD with lysozyme (Fig. S2).

Using an equimolar 80 μM solution of FAD and lysozyme, the initial absorbance is found to be ~10^−3^ (Fig. S2) indicating an initial radical concentration of ~2 μM. The first half life of this signal is ~500 μs. It is reasonable to assume that the radicals recombine with second order kinetics with a diffusion controlled rate constant [S6] of ~10^9^ M^−1^ s^−1^. The radical lifetime can therefore be estimated to be 1/(10^9^ × 2 × 10^−6^) ≈ 500 μs, which is close to the observed value.

Using the same rate constant and the initial radical concentration measured in the Cry experiment (5 μM, see above), the radical lifetime in that experiment would have been 1/(10^9^ × 5 × 10^−6^) ≈ 200 μs if the signal had arisen from reaction of free flavin with accessible amino acid residues in the protein. As reported in the paper, and as shown in Fig. S2, the radical lifetime observed for the Cry sample is approximately 14 ms, i.e. almost *two orders of magnitude slower* than the estimate of 200 μs.

The observed transient absorption signals are thus too long lived to be the result of reactions of free flavin. See **Figure S2**.

[**Figure Legend Fig. S2]** Transient absorption signals at 550 nm recorded for aqueous solutions of 20 μM cryptochrome (purple) and for equimolar mixtures (20, 40 and 80 μM) of FAD with hen lysozyme (blue, orange and red, respectively). The abscissa is time (μs); the ordinate is absorbance. Note the much stronger and much longer lived signal from flavin radicals in the case of the cryptochrome compared to that observed for reactions of free flavin with lysozyme.

***3. Additional Factors***

In general, proteins from the photolyase superfamily are not normally correctly folded and tend to degrade when no FAD is present in the binding pocket. We see no evidence of misfolding or instability in our *gw*Cry1a samples.

In separate experiments it was established that solutions of free flavin (in contrast to the *gw*Cry1 samples) showed no evidence of photoreduction under the same experimental conditions.

(S1) Tsentalovich YP, Lopez JJ, Hore PJ, Sagdeev RZ (2002) Mechanisms of reactions of flavin mononucleotide triplet with aromatic amino acids. Spectrochim Acta A 58: 2043-2050.

(S2) Winder SL, Broadhurst RW, Hore PJ (1995) Photo-CIDNP of amino acids and proteins: effects of competition for flavin triplets. Spectrochim. Acta A 51: 1753-1761.

(S3) Kaptein R, Dijkstra K, Nicolay K (1987) Laser photo-CIDNP as a surface probe for proteins in solution. Nature 274: 293-294.

(S4) Broadhurst RW, Dobson CM, Hore PJ, Radford SE, Rees ML (1991) A photocemically induced dynamic nuclear-polarization study of denatured states of lysozyme. Biochemistry 30: 405-412.

(S5) Hore PJ, Kaptein R (1983) Proton nuclear magnetic resonance assignments and surface accessibility of tryptophan residues in lysozyme. Biochemistry 22: 1906-1911.

(S6) Morozova OB, Yurkovskaya AV, Tsentalovich YP, Forbes MDE et al. (2002) Time resolved CIDNP study of electron transfer reactions in proteins and model compounds. Mol Phys 100, 1187-1195.

(S7) Byrdin M, Eker APM, Vos MH, Brettel K (2003) Dissection of the triple tryptophan electron transfer chain in Escherichia coli DNA photolyase: Trp382 is the primary donor in photoactivation. Proc Natl Acad Sci USA 100: 8676-8681.

(S8) Park HW, Kim ST, Sancar A, Deisenhofer J (1995) Crystal structure of DNA photolyase from Escherichia coli**.** Science 268: 1866-1872.
