# Supplementary figures and images for: Chemical Magnetoreception: Bird Cryptochrome 1a Is Excited by Blue Light and Forms Long-Lived Radical-Pairs
Source: PLoS One. 2007 Oct 31;2(10):e1106. doi: 10.1371/journal.pone.0001106 (PMC2040520; doi:10.1371/journal.pone.0001106)

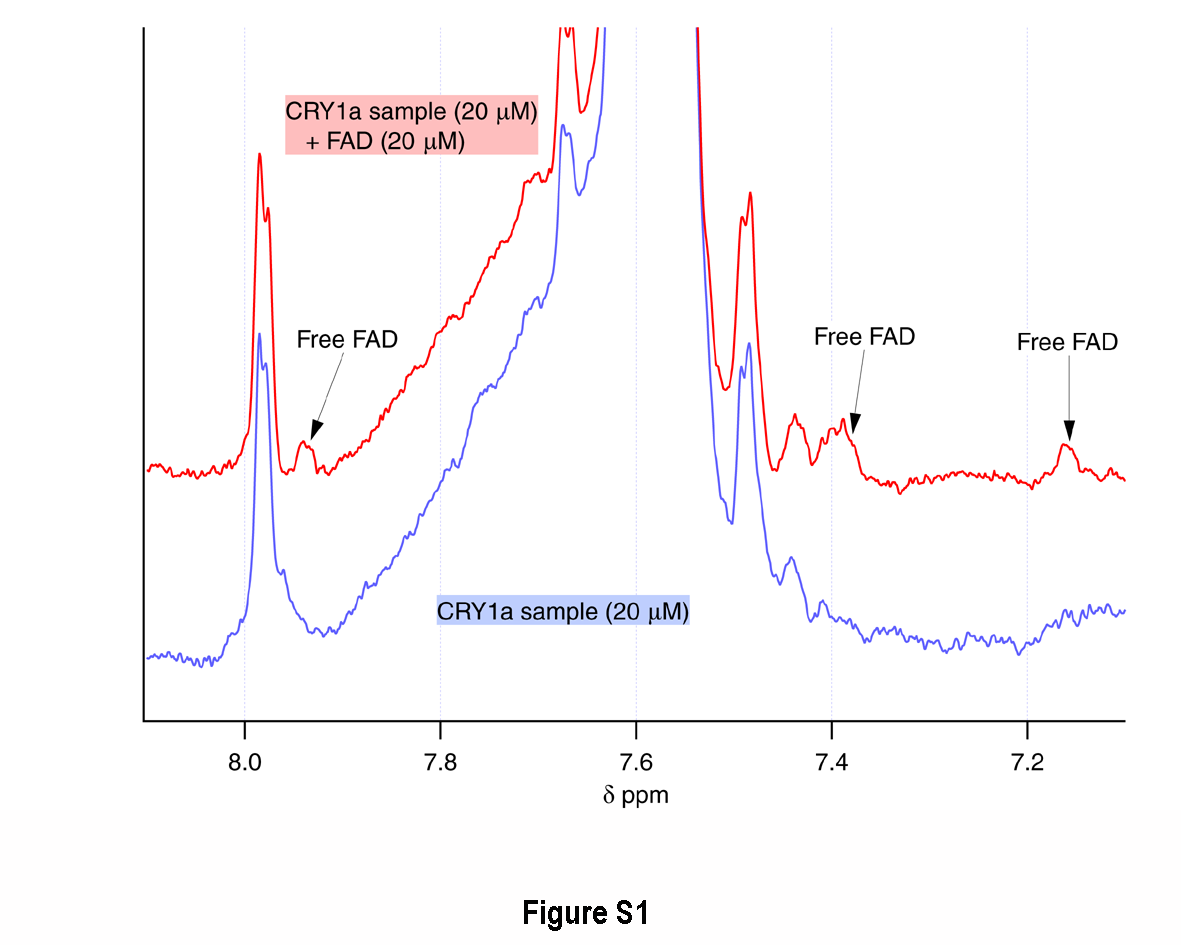

Supplement: Figure S1 — Aromatic region of the 600 MHz 1H NMR spectrum of gwCRY1a protein. Aromatic region of the 600 MHz 1H NMR spectrum of the CRY1a sample used for the transient absorption experiments before (blue) and after (red) addition of 20 µM FAD. Note the additional signals from FAD in the latter spectrum and their absence in the former. (3.63 MB TIF) [file pone.0001106.s002.tif]

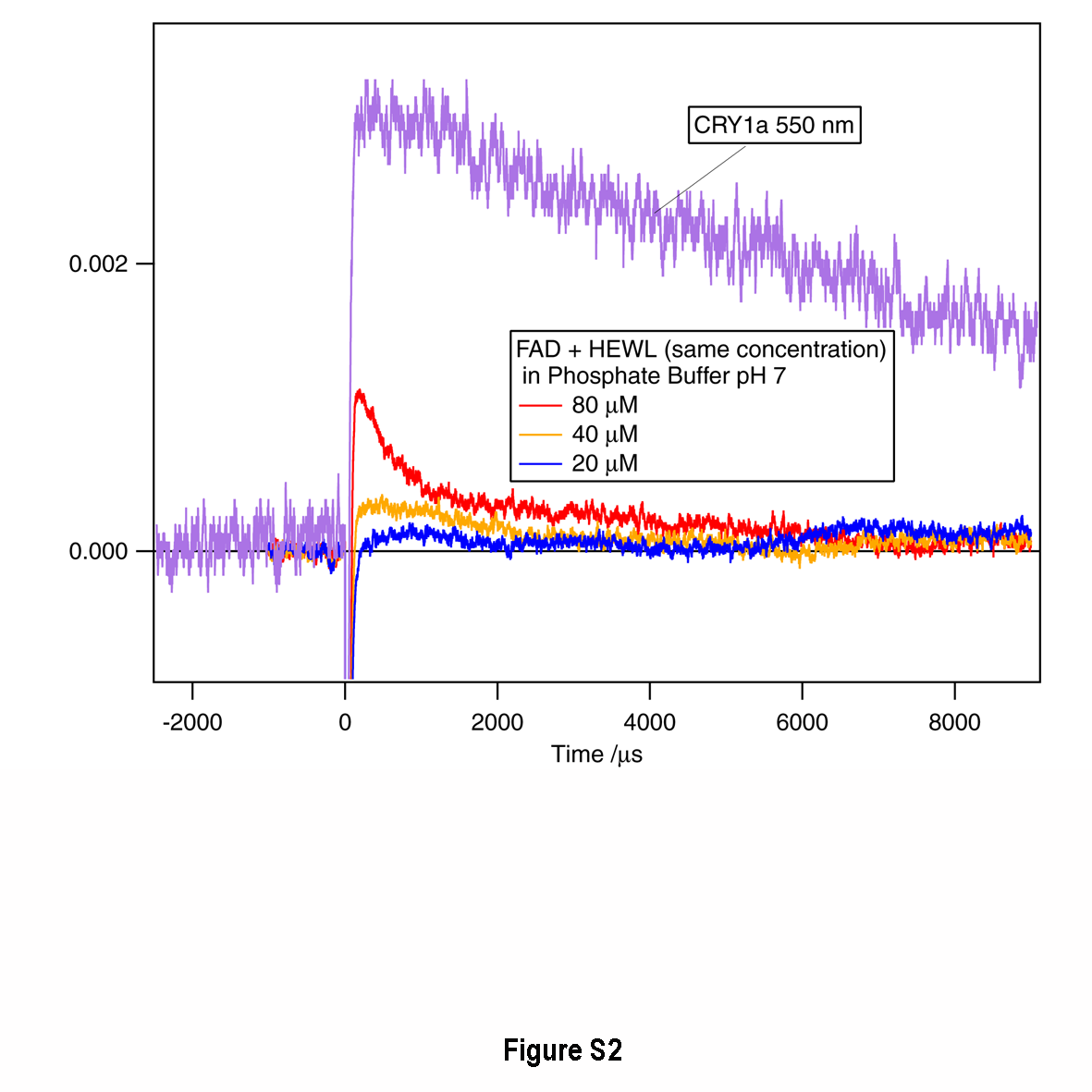

Supplement: Figure S2 — Transient absorption signals at 550 nm for garden warbler cryptochrome and equimolar mixtures of FAD with hen lysozyme. Transient absorption signals at 550 nm recorded for aqueous solutions of 20 µM cryptochrome (purple) and for equimolar mixtures (20, 40 and 80 µM) of FAD with hen lysozyme (blue, orange and red, respectively). The abscissa is time (µs); the ordinate is absorbance. Note the much stronger and much longer lived signal from flavin radicals in the case of the cryptochrome compared to that observed for reactions of free flavin with lysozyme. (4.19 MB TIF) [file pone.0001106.s003.tif]
